# Pyrolysis of Rice Husk for the Production of Bioactive Compounds with Potential in Green Chemistry and Sustainable Agriculture

**DOI:** 10.3390/molecules30132754

**Published:** 2025-06-26

**Authors:** Matheus de Paula Goularte, Ávila Ferreira de Sousa, Camila Cholant, Lucas Romano, Jalel Labidi, André Luiz Missio, Andrey Acosta, Darci Alberto Gatto

**Affiliations:** 1 Postgraduate Program in Materials Science and Engineering (PPGCEM), Federal University of Pelotas, Pelotas 96060-290, Brazil; almatheusgoularte@gmail.com (M.d.P.G.); avilaferreira128@gmal.com (Á.F.d.S.); camila.cholant@gmail.com (C.C.); lucasromano18@outlook.com (L.R.); andre.missio@ufpel.edu.br (A.L.M.); darcigatto@yahoo.com (D.A.G.); 2 Centre of Engineering, Federal University of Pelotas, Pelotas 96060-290, Brazil; 3 Biorefinery Processes Research Group, Chemical and Environmental Engineering Department, Engineering Faculty of Gipuzkoa, University of the Basque Country (UPV/EHU), Plaza Europa 1, 20018 Donostia-San Sebastián, Spain; 4 Postgraduate Program in Materials Science and Engineering (PIPE), Federal University of Paraná, Curitiba 81531-980, Brazil; 5 Postgraduate Program in Forestry Engineering (PPGEF), Federal University of Paraná, Curitiba 81531-980, Brazil

**Keywords:** biomolecules, natural antioxidants, bio-oil, agro-industrial valorisation, bio-crude

## Abstract

The objective of this study was to investigate the chemical composition of the obtained products, as well as the antioxidant activity and bio-stimulant potential of the liquid fractions. The biomass was subjected to pyrolysis in a pilot-scale reactor, followed by distillation of the pyroligneous liquid to separate volatile compounds and enrich bioactive fractions. The samples were analysed by FTIR, TGA/DTG, and GC-MS. Antioxidant activities were assessed through DPPH, FRAP, and total phenolic content assays, while the bio-stimulant potential was evaluated through germination and growth tests of lettuce and arugula seeds. The results showed that the distilled fraction had lower acidity, greater chemical stability, and high antioxidant activity, with the presence of industrially valuable compounds such as methoxylated phenols and furfural. Furthermore, application of the distilled liquid at 0.1% concentration stimulated early seedling development—especially in arugula—while higher concentrations demonstrated inhibitory effects. These findings show that distillation of pyroligneous liquid is an effective strategy to enhance its bioactive properties, enabling its use as a natural antioxidant and plant bio-stimulant.

## 1. Introduction

The thermal conversion of rice husk can be carried out through processes such as combustion, gasification, and pyrolysis—the latter being one of the most versatile, as it enables the production of three main products: biochar, permanent gases, and bio-oil, also known as pyroligneous acid or wood vinegar [1,2]. This liquid consists of a complex mixture of organic compounds, such as phenols, organic acids, ketones, and furans, many of which are known to exhibit biological activity [3,4]. Research indicates that the chemical composition of pyroligneous acid can significantly influence its bioactivity, especially in applications such as agricultural bio-stimulants, cosmetics, and natural pesticides [5,6]. Recent reviews have reinforced this multifunctionality by highlighting the presence of over 200 compounds in pyroligneous acid, including phenols, aldehydes, and carboxylic acids, which contribute to its antimicrobial, antioxidant, and plant-stimulating properties [7,8].

The growing global demand for sustainable agricultural practices has intensified interest in low-impact inputs. In this context, bio-stimulants have gained prominence for their ability to stimulate natural physiological processes in plants, enhancing nutrient uptake, growth, and resistance to abiotic stress, without causing adverse environmental effects [9]. Pyroligneous acid derived from rice husk has been identified as a promising biostimulant, capable of improving seed germination and root development at specific concentrations, depending on its chemical composition and pyrolysis conditions [10,11].

However, the direct application of pyroligneous acid may be limited due to its high acidity and chemical instability. Fractional distillation techniques have been widely employed to overcome these limitations, allowing for the selective separation of volatile compounds and the production of more purified and bioactive fractions. Although distillation is a well-established process for improving the chemical quality of pyroligneous acid, there are still gaps in understanding how this process affects the bioactivity of its fractions, particularly regarding their antioxidant capacity and bio-stimulant potential [1].

The composition and properties of pyroligneous acid vary according to the biomass used and the pyrolysis parameters, such as temperature and residence time. Therefore, detailed characterisation of the resulting fractions is essential to understand their potential applications. Analytical techniques such as Fourier Transform Infrared Spectroscopy (FTIR) and Gas Chromatography–Mass Spectrometry (GC-MS) are key tools for identifying the compounds present and assessing their possible bioactive functions [2,4].

Rice husk, an abundant agricultural residue rich in lignocellulosic and siliceous materials, has been increasingly valorised through pyrolysis for energy and material recovery [12,13]. While many studies have focused on the fuel properties of bio-oils obtained from biomass [14], fewer works have explored the biological potential of the condensed liquid fractions—particularly the pyroligneous liquid (PL), which contains a wide range of oxygenated compounds. Recent research has reported the presence of phenolic, carboxylic, and furan-based compounds in PL with promising antioxidant or plant growth-regulating effect [11,15,16]. However, little is known about the relationship between fractionation, chemical composition, and bioactivity in PLs derived from rice husk. A study by Kuo et al. [17] demonstrated the feasibility of producing pyroligneous acid from rice husk using a combustion-based system, but without exploring the chemical refinement or biological potential of the resulting fractions, indicating a knowledge gap that is addressed in the present work [17].

Thus, the thermochemical valorisation of rice husk represents a sustainable alternative for generating bioproducts with industrial and agricultural applications, fostering the circular economy and contributing to environmental impact reduction. Rice husk accounts for up to 20% of harvested rice by weight, and more than 150 million tons are generated annually worldwide [18]. Improper disposal methods—such as open-air burning or uncontrolled dumping—release significant amounts of methane and carbon dioxide, contributing to climate change and local pollution [19,20]. In contrast, slow pyrolysis converts organic carbon into value-added products such as pyroligneous liquid and biochar, reducing greenhouse gas emissions and enabling the recovery of chemical compounds with functional properties [21,22]. Moreover, understanding the chemical transformations resulting from pyroligneous acid distillation and their effects on biostimulant activity opens new perspectives for using this by-product as an innovative agricultural input [23,24]. The outcomes of this work directly contribute to four Sustainable Development Goals (SDGs): SDG 9 (Industry, Innovation and Infrastructure), SDG 12 (Responsible Consumption and Production), SDG 13 (Climate Action), and SDG 15 (Life on Land) [25]. Therefore, the objective of this study was to produce and characterize the bioactive potential of liquid fractions obtained through slow pyrolysis of rice husk, aiming to explore their chemical composition and potential applications in agriculture and green chemistry. The novelty of this work lies in integrating a low-cost pyrolysis route with bioactivity evaluation (antioxidant and biostimulant) of condensed fractions, particularly highlighting the role of distillation in enhancing bioactive compound concentration.

## 2. Results and Discussion

### 2.1. Slow Pyrolysis Process and Characterization

**Figure 1:**
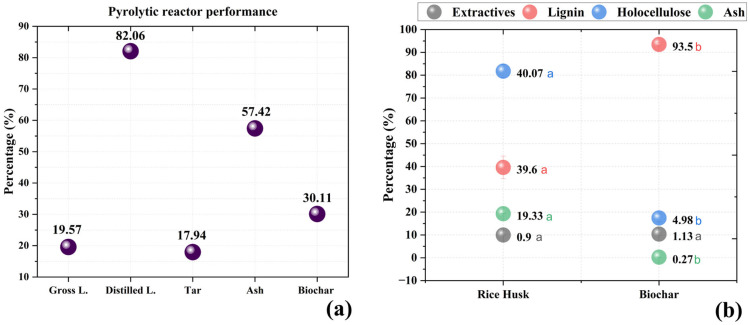
(**a**) Yield analysis of pyrolytic fractions; (**b**) Comparison of the chemical composition of raw rice husk and biochar; means followed by the same letter in the row do not differ significantly from each other according to Tukey’s test (*p* < 0.05).

The analysis of the yields obtained from biomass pyrolysis revealed essential information about the efficiency of the process and the distribution of the generated products. In the present study, the average yields were 19.57% for crude pyroligneous liquid (LB), 30.11% for biochar, and 11.25% for ash (Figure 1). These values fall within the expected range for slow pyrolysis processes, which typically result in more efficient conversion to biochar, whereas fast pyrolysis favours the formation of gases and bio-oil [16].

Previous studies have shown that the yields of pyroligneous liquids vary according to temperature and biomass type. Pimenta et al. [26] reported that, in the pyrolysis of residual biomass, liquid yields may range between 15% and 30% at lower temperatures (300–500 °C), while temperatures above 600 °C promote greater conversion to gases, reducing the liquid fraction. In this study, the values found are consistent with this range, indicating that the process employed favoured the production of balanced liquid fractions alongside residual biochar. When comparing the obtained yields with other pyrolysis reactors, it is observed that the results are similar to those reported by Hernández-Ibáñez et al. [27], who found average yields of 22–28% for pyroligneous liquids from the pyrolysis of residual wood and 35–40% for biochar. The variation observed in biochar yield in this study (17.88% to 42.33%) may be related to the heterogeneity of the biomass used and differences in heating rates and residence time [28]. Fractional distillation of the crude pyroligneous liquid resulted in an average yield of 82.06% for the distillate and 17.94% for the soluble tar. This result aligns with values reported for pyroligneous acids from different sources, where fractional distillation can achieve 70–90% recovery of the volatile fraction [29]. However, the chemical composition of the distilled liquid may vary depending on the biomass and distillation parameters. Studies indicate that soluble tar tends to concentrate heavier phenolic and aliphatic compounds, while the distillate contains lighter volatile fractions, such as alcohols, organic acids, and ketones [27]. Distillation efficiency can also be assessed in comparison with other sources of pyroligneous liquids. Loo et al. [30] reported yields of 75–85% for distilled fractions of pyroligneous acids from *Rhizophora apiculata*, while Pimenta et al. [26] observed similar values in extracts from forest biomass, reinforcing that the results obtained in this study are within the expected range for well-controlled distillation processes.

**Figure 2:**
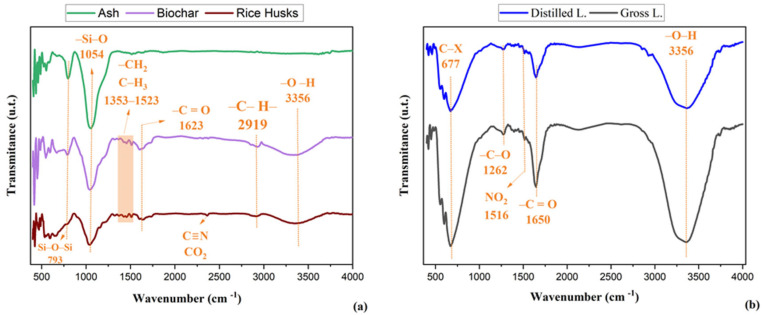
(**a**) Infrared spectrum of raw rice husk, biochar, and ash; (**b**) Infrared spectrum of the pyroligneous liquid from rice husk.

Figure 1b illustrates the chemical composition of raw rice husk and the biochar produced through slow pyrolysis, providing a clear visualisation of the changes in chemical constituents after pyrolysis. A significant increase in lignin and holocellulose content is observed, while ash content decreased drastically. After pyrolysis, a relative increase in lignin and holocellulose content was observed in the biochar compared to raw rice husk. This phenomenon is attributed to the selective thermal decomposition of biomass components. During pyrolysis, hemicellulose and cellulose are more susceptible to thermal degradation, whereas lignin, due to its complex aromatic structure, shows greater resistance, resulting in its relative concentration in biochar [31,32]. Studies have shown that lignin possesses a robust three-dimensional structure, making it more resistant to decomposition compared to polysaccharides [33]. Additionally, holocellulose, which comprises cellulose and hemicellulose, also undergoes thermal degradation; however, part of the cellulose may resist the process, contributing to the relative increase observed in the biochar. The thermal resistance of cellulose is attributed to its crystalline structure, which provides greater stability at elevated temperatures [34,35]. Extractives, composed of low thermal stability compounds, tend to volatilise during pyrolysis. However, the residual presence of these compounds in biochar may be explained by the formation of new compounds during the process, which remain in the final material. During thermal degradation, volatile compounds may recombine or condense within the biochar structure, forming new extractives [36]. As for ash content, a significant reduction was observed in the biochar compared to raw rice husk, as shown in Figure 2. This phenomenon can be attributed to the volatilisation of minerals during pyrolysis or the transformation of mineral components into gaseous forms [31].

The modifications in chemical composition between raw rice husk and biochar have significant implications for their applications. The relative increase in lignin and holocellulose in biochar suggests a more stable and carbon-rich structure, which may be beneficial for applications such as soil conditioning, improving nutrient retention and soil structure [37]. Studies show that lignin contributes to the recalcitrance of biochar, increasing its persistence in soil and enhancing agronomic benefits [11]. On the other hand, the residual presence of extractives may influence the chemical properties of biochar, affecting its interaction with soil and microorganisms. The reduction in ash content indicates a lower mineral concentration, which may be relevant depending on the intended application of the biochar. The mineral composition of biochar can affect its adsorption capacity and its effectiveness as an agricultural amendment [31].

Figure 2a,b presents the FTIR spectra of the raw rice husk, biochar and ash samples, and of the raw pyroligneous liquid (LB) and distilled pyroligneous liquid (LD) respectively, showing significant chemical differences between the materials.

The band observed at approximately 3338 cm^−1^ in both biochar and raw rice husk is associated with O–H stretching vibrations in alcohols, phenols, and residual water. In biochar, this band indicates the retention of phenolic or alcoholic functional groups, as well as the possible presence of adsorbed water on the surface of the carbonised material [31]. The band at 2363 cm^−1^, present in raw husk and associated with C≡N stretching in nitriles or dissolved CO_2_ vibrations, is not observed in biochar or ash, indicating these compounds were eliminated or transformed during the carbonisation process [31,35]. The band at 1598 cm^−1^, observed in both biochar and raw husk, is associated with C=C stretching in aromatic rings, indicating the presence of lignin and other aromatic compounds. Its persistence in biochar suggests that part of the aromatic structures is retained during pyrolysis, contributing to the carbon structure of the material [38,39]. The band at 1423 cm^−1^, also present in both samples, corresponds to CH_2_ or CH_3_ bending, related to cellulose, hemicellulose, and lignin. Its continued presence in biochar indicates the retention of aliphatic and aromatic structures after carbonisation, preserving characteristics of the original biopolymers [40,41].

In the ash spectrum, the band at 1051 cm^−1^ is linked to Si–O stretching in silicates, indicating the concentration of mineral components after complete carbonisation [42,43]. In biochar, the band at 1043 cm^−1^, associated with C–O–C stretching in ethers and Si–O in silicates, suggests the formation or concentration of these functional groups after pyrolysis. In raw husk, the band at 1034 cm^−1^ is attributed to C–O–C stretching in ethers and C–O in alcohols, phenols, or carboxylic acids, reflecting its complex organic matrix [44,45]. The bands at 798 cm^−1^ and 792 cm^−1^, observed in ash and biochar, respectively, are associated with Si–O–Si stretching in silicates, indicating their persistence and concentration in the ash [46]. In raw husk, only a shoulder is observed in this region, suggesting lower silicate content or less defined interaction within the lignocellulosic matrix [30].

Figure 2b shows distillation promotes the removal of volatile compounds and may increase the purity of specific functional groups [2,47]. The trough at 1645 cm^−1^ is related to C=C bonds in aromatic compounds and C=O stretching in ketones, aldehydes, and carboxylic acids, indicating phenolic or carbonyl presence. Its observation suggests a lower concentration of these compounds in the crude liquid compared to the distilled fraction [47,48]. The band at 1650 cm^−1^ is attributed to the stretching vibrations of conjugated carbonyl groups (C=O), which may arise from ketones, aldehydes, and carboxylic acids, as well as C=C stretching of aromatic structures. This interpretation aligns with the GC-MS data, which confirmed the presence of oxygenated compounds such as furfural, 1-hydroxy-2-butanone, and phenolic derivatives in both fractions. At 1515 cm^−1^, the decrease in band intensity indicates a reduction in aromatic compounds such as phenols and benzene derivatives, or NO_2_ groups from nitro compounds—typical behaviour due to lower concentration in the crude liquid [49,50]. The band at 1272 cm^−1^, associated with C–O stretching in alcohols, ethers, or carboxylic acids, also shows a trough, suggesting reduced levels of these functional groups in the crude pyroligneous liquid [43]. The bands at 671, 599, 553, 450, and 420 cm^−1^ show a decrease in compounds with C–H bonds in aromatic rings or C–X (halogen) bonds, indicating a reduced presence in the crude liquid [51,52].

**Figure 3:**
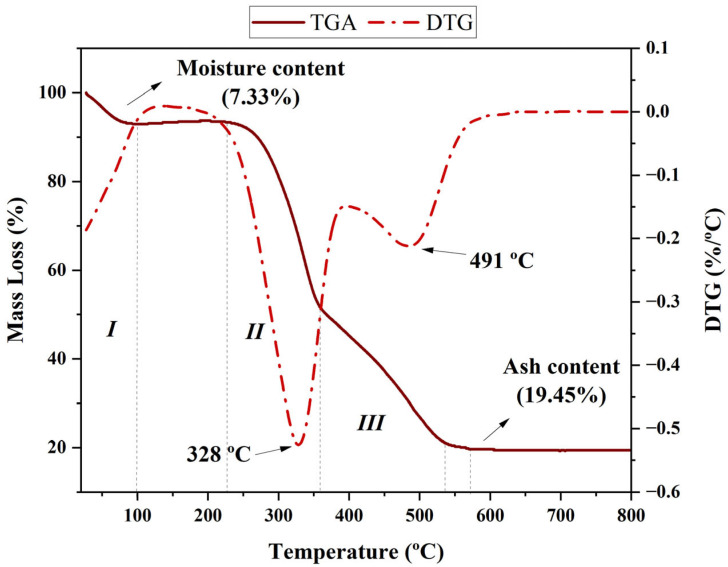
Thermogravimetric analysis (continuous line) and derivative thermogravimetric analysis (dotted line) of rice husk.

The thermal behaviour of the studied biomass shows three distinct regions of mass loss, which are characteristic of lignocellulosic materials (Figure 3).

According to Figure 3 (I), in the first region, between 30 and 150 °C, a mass loss of approximately 7.33% is observed, related to the removal of moisture and light volatile compounds. This behaviour is typical of lignocellulosic biomass and is associated with the evaporation of free water and volatile compounds bound by weak intermolecular forces [53]. The second region, between 250 and 400 °C, corresponds to the thermal degradation of the main organic constituents of rice husk. Hemicellulose decomposition occurs between 200 and 350 °C, while cellulose exhibits significant degradation between 300 and 400 °C, resulting in the formation of volatile compounds such as CO, CO_2_, and light organics [54]. Lignin, in turn, shows a more distributed decomposition behaviour, occurring over a broader temperature range from 250 to 500 °C due to its complex and highly aromatic structure [55]. The DTG curve peaks at 328 °C and 491 °C indicate the maximum decomposition rates of cellulose and lignin, respectively, revealing that the greatest thermal conversion of biomass occurs in this interval. As shown in Figure 3, these thermal transitions occur within well-defined temperature ranges, with cellulose decomposing rapidly, while lignin degrades more slowly, contributing to biochar formation. The third region, above 500 °C, is characterised by residual carbonisation and ash formation. The fixed residue observed after this stage corresponds to 19.45% of the initial mass, indicating a high ash content, typical of biomass with high silica content, such as rice husk. This aspect is particularly relevant, as silica may influence the efficiency of the pyrolysis process and the reactivity of the resulting biochar [56]. The presence of alkali and alkaline earth metals in the ash can catalyse secondary reactions, reducing bio-oil production and favouring biochar formation, thus making this residue a valuable material for applications such as contaminant adsorption and agricultural soil improvement [56,57].

### 2.2. Liquid Fraction Analysis

**Figure 4:**
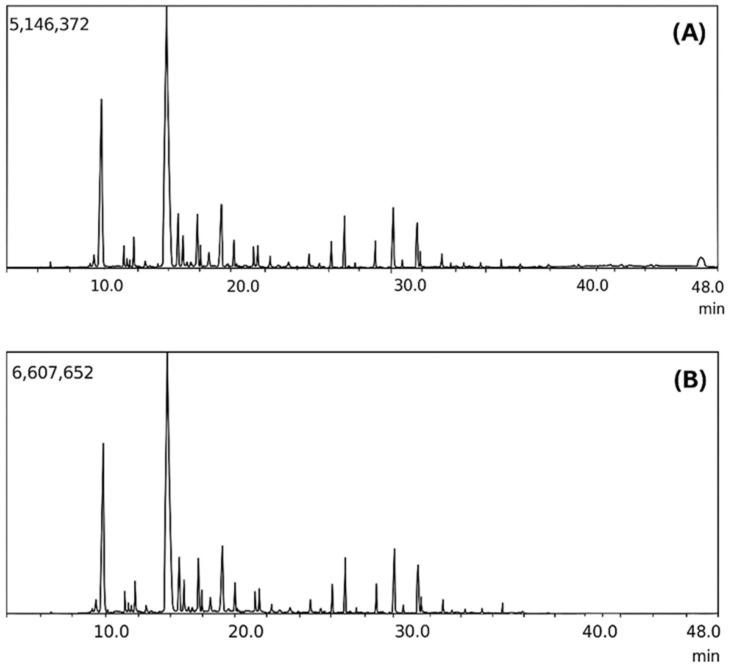
(**A**) Chromatogram of crude pyroligneous liquid; (**B**) Chromatogram of distilled pyroligneous liquid.

The distillation of the pyroligneous liquid caused significant changes in its chemical composition, altering both its antioxidant properties and its bioactivity (Figure 4).

Volatile compounds, particularly organic acids, were removed during distillation, while certain phenolic and aromatic compounds were preserved or enriched. This change had a direct impact on the characteristics of the distilled pyroligneous liquid (LD), making it less acidic and more chemically stable. Additionally, one of the major compounds identified in LD, 1-Hydroxy-2-butanone, is an important intermediate in the synthesis of pharmaceuticals used in the treatment of tuberculosis, suggesting potential biomedical applications [4]. The chemical composition of the crude pyroligneous liquid (LB), presented in Table 1, revealed the predominance of acetic acid (52.84%), followed by 2-Propanone, 1-hydroxy- (11.69%), and formic acid (2.85%), along with other phenolic and oxygenated compounds. The distillate column in Table 1 shows that the distillation process significantly altered this composition, leading to the complete elimination of acetic and formic acids—highly volatile compounds that contribute to the elevated acidity of LB. The removal of these acids directly impacts the chemical and biological stability of LD, as the reduction in acidity favours the preservation of phenolic and aromatic compounds that could otherwise degrade under harsher conditions [58,59].

Another relevant point was the change in the concentration of certain oxygenated compounds. 2-Propanone, 1-hydroxy (acetol), which was already present in the crude pyroligneous liquid (LB), was found in a higher proportion in the distilled liquid (LD), as shown in Table 1. This increase suggests that the distillation process not only removes volatile compounds but may also concentrate fewer volatile substances. However, its high presence in LD does not significantly contribute to its antioxidant properties, since ketones and secondary alcohols like this compound have low electron-donating capacity and do not actively neutralise free radicals [60].

One of the most expressive changes observed in Table 1 was the high concentration of 1-Hydroxy-2-butanone in LD (51.53%), a compound absent in LB but which emerged as the main constituent after distillation. This compound has been widely studied in the pharmaceutical industry as an intermediate in the synthesis of antituberculosis drugs, suggesting that LD may have applications beyond those traditionally explored for pyroligneous liquids [4,58]. Its presence in significant quantities indicates that the distillation process not only purifies the liquid but also promotes the selective enrichment of specific bioactive compounds, potentially adding value to LD in areas such as biotechnology and chemical synthesis.

Although the major compounds in LD do not exhibit significant antioxidant properties, the presence of minor compounds may explain the differences observed between the analysed fractions. Phenol, 2-methoxy- (guaiacol), identified in LD at a concentration of 3.46% (Table 1), is known for its antioxidant activity, as its conjugated phenolic structure facilitates free radical stabilisation [15]. Its persistence after distillation indicates that LD may retain relevant bioactive properties, supporting its use as a natural preservative agent in various industrial applications. In addition to guaiacol, compounds such as Carbamic acid, phenyl ester (3.42%), and furfural (2.92%) also remained in the distilled fraction, suggesting that distillation can act as a selective concentration method for bioactive molecules [34], a semi-quantitative comparison based on the relative peak areas from the GC-MS data (Table 1), highlights the compositional shifts caused by distillation. For instance, acetic acid, which represented 52.84% of the crude liquid (LB), was completely removed in the distilled fraction (LD), confirming the effective separation of highly volatile acids. In contrast, 1-hydroxy-2-butanone became the most abundant compound in LD, accounting for 51.53% of the chromatographic area, while being virtually absent in LB. Methoxylated phenols, such as 2-methoxyphenol (guaiacol), also showed increased relative abundance in LD (from 2.5% to 3.46%), suggesting enrichment of bioactive aromatic compounds. This selective concentration underscores the efficiency of distillation in tailoring the chemical profile of pyroligneous liquid for specific applications.

In the crude pyroligneous liquid, the predominant compounds were acetic acid (52.84%), furfural (1.37%), formic acid (2.85%), and propionic acid (2.49%). Furthermore, the study revealed enhanced antioxidant performance and significant bioactivity effects of the distilled fraction, which are presented in the subsequent sections. These compounds are widely recognised for their interaction with plant physiological processes, potentially influencing root growth regulation [15,34,61,62]. Acetic acid, for example, may affect metabolic pathways related to auxin biosynthesis, key phytohormones involved in root development [63,64]. Furfural, a compound derived from polysaccharide degradation, has also been associated with positive effects on plant metabolism, especially in promoting abiotic stress resistance [6,61]. These findings are consistent with the studies of Mathew and Zakaria [7], who highlighted the biological potential of phenolic and oxygenated compounds present in pyroligneous acid. Kuo et al. [17] reported the production of rice husk-derived pyroligneous acid and its use as a plant growth promoter, although without distillation refinement. More recently, Candido et al. [8] reinforced the multifunctionality of such liquids, linking their phenolic profile to antioxidant activity and root development in crops.

In the distilled fraction (Table 1), a relative increase in the concentration of 1-hydroxy-2-butanone (51.53%) was observed, along with a reduction in the presence of phenols and organic acids compared to the crude fraction. Furfural remained present, although in a lower concentration (2.92%), while methoxylated phenolic compounds such as 2-methoxyphenol and 2-methoxy-4-methylphenol were partially preserved in the distilled fraction. The presence of these compounds suggests that, despite the removal of higher molecular weight phenols, some thermally stable bioactive compounds remain after the distillation process [62,65].

The carbonyl-related bands observed in the FTIR spectra of the distilled fraction (LD) can be attributed not only to residual carboxylic acids but also to other carbonyl-containing compounds such as ketones and aldehydes. This interpretation is supported by the GC-MS results, which identified compounds like 1-hydroxy-2-butanone and furfural in LD. These species contribute to the C=O absorption band in the region around 1700 cm^−1^, even when total acid content is low [66,67]. These findings are crucial to understanding the biostimulant potential of the liquid fractions, providing a basis for future studies on the mechanism of action of these compounds in plant rooting. GC-MS analysis confirmed the presence of compounds known for their bioactivity, highlighting the importance of the chemical composition of pyroligneous liquid for potential applications in plant development [29,68].

### 2.3. Antioxidant Activity of the Liquid Fractions

The evaluation of the antioxidant activity of the pyroligneous liquid fractions (LB and LD) and the tar revealed significant differences that can be attributed to variations in their chemical composition. As shown in Table 2, the distilled fraction (LD) presented an EC_50_ value in the DPPH assay of 10.98 ± 2.30 μg/mL, indicating high antioxidant capacity. In contrast, the tar showed an EC_50_ of 216.78 ± 40.10 μg/mL, while the crude pyroligneous liquid (LB) recorded a value of 7.881 ± 3.437 μg/mL, indicating that both LB and LD exhibit significantly higher antioxidant activities compared to the tar.

The explanation for this difference is directly related to the chemical composition of each fraction, as previously discussed in Table 2. The crude pyroligneous liquid (LB) exhibited a high content of total phenolic compounds (713.72 ± 6.17 mg/mL), while the distilled liquid (LD), although subjected to distillation, still retained a significant level (708.38 ± 1.37 mg/mL). These values were obtained by the Folin–Ciocalteu method, which measures the total phenolic hydroxyl content, and may include compounds not detected by GC-MS. Tar, on the other hand, despite having a high concentration of aromatic and polyaromatic compounds [69], showed a slightly higher total phenolic content than LB (729.25 ± 7.55 mg/mL) but exhibited lower antioxidant activity in both the DPPH and FRAP assays. This indicates that not all phenolic compounds present in tar are highly effective in neutralising free radicals, which may be attributed to their chemical structure and the highly non-polar environment of this fraction.

The lower antioxidant capacity of tar may be explained by the fact that, although it contains phenolic compounds, many of them are associated with highly condensed and polyaromatic structures, which hinder the availability of hydroxyl groups to act in free radical neutralisation [15,70]. Unlike the liquid fractions, where phenolics are dissolved in a more polar medium and are more readily accessible for electron donation, in tar, many compounds may be stabilised in graphitised structures, reducing their antioxidant efficacy. These findings reinforce that, although the crude pyroligneous liquid (LB) exhibited slightly higher antioxidant activity than the distilled fraction (LD) in the DPPH assay, fractionation does not necessarily reduce the functional value of the liquid. Instead, it enables the separation and concentration of specific compounds with different bioactivities. While LD retained sufficient antioxidant potential, as indicated by its low EC_50_ and high FRAP value, it also demonstrated superior biostimulant performance in seed germination assays. This highlights that the fractionation process may be advantageous depending on the desired application—whether targeting antioxidant function or plant-growth promotion.

The difference observed between LB and LD is also chemically interesting. LB contained a high concentration of acetic acid (52.84%), which was completely removed during distillation, thus reducing the acidity of the distilled fraction. Moreover, distillation led to the concentration of compounds such as 1-hydroxy-2-butanone (51.53%), which does not exhibit significant antioxidant properties but may contribute to other biochemical functions, such as its use in the synthesis of anti-tuberculosis drugs [4,58]. On the other hand, relevant antioxidant compounds such as 2-methoxyphenol (3.46%) and carbamic acid, phenyl ester (3.42%) remained in the distilled fraction, explaining its high antioxidant activity even after the removal of organic acids.

Another relevant aspect of the tar fraction is its relatively low antioxidant activity in the FRAP assay (6.08 ± 0.25 μM Fe^2+^/g), suggesting that the compounds present in this fraction have lower reducing power compared to the liquid fractions (LB and LD). This supports the hypothesis that tar contains stabilised aromatic compounds that do not actively participate in electron transfer processes, unlike the phenolic compounds present in LB and LD.

Previous studies on bio-oils derived from biomass pyrolysis suggest that antioxidant activity is strongly correlated with the presence of light phenolic compounds and volatile organic acids [58,59]. Fractions of bio-oil rich in organic acids and phenolic compounds have been reported to exhibit high antioxidant activity, whereas those containing polycyclic compounds tend to show low efficiency in free radical scavenging, as discussed in the literature [47]. These findings align with our results, in which LD, despite the removal of organic acids, retained active phenolic compounds, ensuring its antioxidant activity. The antioxidant activity observed in both crude and distilled fractions is largely attributed to the presence of phenolic compounds with conjugated structures, such as 2-methoxyphenol (guaiacol). This compound acts as an effective radical scavenger by donating hydrogen atoms and stabilizing reactive species through resonance delocalization, showing additional antimicrobial and oxidative stress modulation effects in fungal systems [71]. Furfural, another abundant compound in the distilled fraction, also contributes to antioxidant and biostimulant performance due to its furan ring and aldehyde group, which enhance reactivity and may promote redox homeostasis in plants [72]. Additionally, ketones like 1-hydroxy-2-butanone can act synergistically with phenolics by improving antioxidant persistence over time [73]. These interactions may be further stabilized by hydrogen bonding and hydrophobic interactions with other biomolecules, as commonly reported in protein–polyphenol systems [74].

Furthermore, Loo et al. [30] evaluated pyroligneous acid produced from *Rhizophora apiculata* and observed that phenolic content is not the sole determinant of antioxidant activity. According to the authors, the chemical structure and polarity of the medium directly influence the availability of antioxidant compounds, explaining why tar, despite its high phenolic content, exhibited lower antioxidant efficiency in the present study [63].

### 2.4. Evaluation of Plant Growth Stimulation

The evaluation of the biostimulant potential of pyroligneous liquid on lettuce (*Lactuca sativa*) and arugula (*Eruca sativa*) seeds revealed significant variations among the treatments, indicating that different concentrations and fractions of the liquid influence seedling growth in distinct ways.

**Figure 5:**
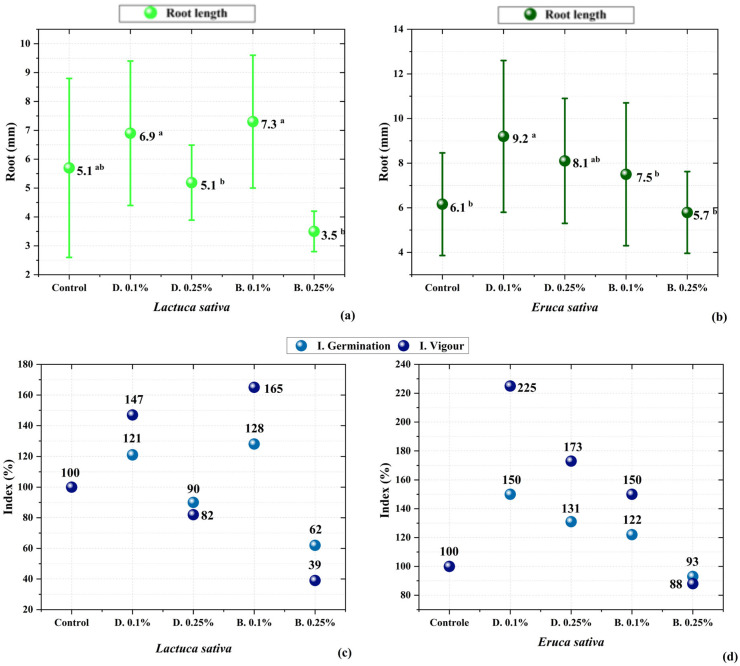
Growth response among cultivars and treatments: (**a**) *Lactuca sativa*; (**b**) *Eruca sativa*. Effect of pyroligneous liquid treatment concentrations on germination, relative elongation, and seed vigour of (**a**) lettuce and (**b**) arugula. Means followed by the same letter in the row do not differ significantly from each other according to Tukey’s test (*p* < 0.05). (**c**,**d**) Germination index (GI) calculated based on the relative germination rate and root length in comparison to the control. No statistical test was applied to these figures due to the formula-derived nature of the index.

Figure 5a,b presents the visual results of seed germination under different treatments, showing the effect of pyroligneous liquid concentrations on seedling development. It was observed that the distilled fraction at a 0.1% concentration promoted initial growth by stimulating radicle elongation, indicating a biostimulant effect. In contrast, increasing the concentration to 0.5% resulted in total inhibition of germination, suggesting phytotoxic effects on the seeds [34,64].

Moreover, the average values of radicle length reinforce this trend, demonstrating the stimulatory effect promoted by the 0.1% concentration. At higher concentrations of pyroligneous liquid, a complete inhibition of growth was observed, clearly indicating phytotoxicity and reinforcing the importance of dilution for bioactivity applications [29,64,65,75]. Among the evaluated species, *Eruca sativa* (arugula) showed greater tolerance to the treatment, maintaining development even under adverse conditions, whereas *Lactuca sativa* (lettuce) exhibited higher sensitivity to the compounds present in the pyroligneous liquid.

Germination index, elongation, and vigour indices were calculated to quantify the impact of the crude and distilled pyroligneous liquid fractions on the tested seeds. The results are shown in Figure 5c,d, where it can be seen that the distilled fraction at 0.1% exhibited the highest indices for both cultivars. The crude fraction, even at low concentrations, did not present such a clear stimulation pattern, possibly due to the presence of inhibitory compounds in its composition [62,66,76].

The observed effects can be explained by the presence of bioactive organic compounds identified through GC-MS analysis (as previously discussed). As shown in Figure 3, lettuce exhibited a more sensitive response to the compounds in the pyroligneous liquid, while arugula demonstrated greater tolerance, reflected in less variation between treatments [65,66].

Similar results were reported by Silva et al. [77], in which liquid fractions derived from biomass pyrolysis were tested on different cultivars, showing that more purified (distilled) fractions tend to exhibit higher biostimulant potential due to the removal of inhibitory compounds. Furthermore, studies such as Wei et al. [70] suggest that phenolic compounds and organic acids present in pyroligneous liquid may modulate root development depending on the applied concentration.

These findings suggest that pyroligneous liquid may be a viable alternative for sustainable agricultural applications, provided it is used at appropriate concentrations. The use of the distilled fraction at 0.1% proved promising as a natural biostimulant for early seedling development [77,78]. These results align with previous findings from Mathew and Zakaria [7], who demonstrated that phenolic compounds such as guaiacol and cresols in pyroligneous acid may mimic auxin-like effects, promoting root growth [7]. Similarly, Candido et al. [8] observed that fractionated pyroligneous acid enhanced early root elongation, which was attributed to the enrichment of low-molecular-weight phenols and carboxylic acids after distillation. Additionally, Kuo et al. [17] reported improved seedling vigour and root density following the application of rice husk-derived pyroligneous liquid at low concentrations.

## 3. Materials and Methods

### 3.1. Raw Materials

Rice husks were supplied by the company Nelson Wendt Cia LTDA., located in Pelotas, RS, Brazil. For the preparation of the samples submitted to chemical analysis, the husks underwent particle size reduction using a Willey-type knife mill (Marconi brand, Piracicaba, SP, Brazil). The material was then sieved, and the fraction retained on the 60 mesh screen was used, following the TAPPI T 257 standard for sampling and preparation of wood for analysis [79].

### 3.2. Slow Pyrolysis Process

The process was carried out in a pilot-scale stainless-steel reactor with a total capacity of 3.5 L. A mass of 1.5 kg of raw rice husk was placed into the ignition chamber, and biomass ignition was initiated by a resistance coil for 15 min. After this period, thermal decomposition progressed autonomously. The process was conducted within a temperature range of 623.15 K to 723.15 K (350–450 °C), with a heating rate of 0.5 K/min, and a total residence time of 40 min. The pyroligneous liquid was continuously collected during this period, particularly between 298.15 K and 303.15 K (25–30 °C) through condensation. After the reactor cooled to room temperature, the biochar and ash were collected for characterization.

### 3.3. Chemical Characterisation of Rice Husk

The extractive content of the raw husk and the resulting biochar was determined using organic solvents, following TAPPI standards T 204 cm-17, with benzene replaced by toluene [80]. The insoluble lignin content was measured using the Klason method, in accordance with TAPPI standards T 222 om-02 [81]. Holocellulose content was determined following TAPPI T 9 Wd 75 procedures [82], enabling the comparison of chemical composition and structural changes before and after carbonisation. Ash content was determined according to ASTM D1762 [83].

### 3.4. Fourier-Transform Infrared Spectroscopy (FTIR)

The functional groups present in solid and liquid samples were analysed by Fourier-transform infrared spectroscopy using an attenuated total reflectance accessory (ATR-FTIR) coupled to a Jasco 4100 spectrometer (JASCO Corporation, Tokyo, Japan). A total of 32 scans were performed across the spectral range of 450 to 4000 cm^−1^, with a resolution of 4 cm^−1^.

### 3.5. Thermal Characterisation

The thermal decomposition of rice husk was assessed by thermogravimetric analysis (TGA) using a Navas TGA-1000 instrument (Navas Instrumentos, Piraciaba, SP, Brazil). Approximately 10 mg of dried rice husk sample was placed in a platinum crucible and heated from room temperature to 800 °C at a constant heating rate of 10 °C/min under a nitrogen atmosphere with a flow rate of 50 mL/min to ensure an inert environment. The analysis was conducted in triplicate to confirm repeatability. Thermogravimetric (TG) and derivative thermogravimetric (DTG) curves were recorded, and decomposition stages were interpreted based on mass loss events.

### 3.6. Distillation of Pyroligneous

The crude pyroligneous liquid was transferred to a distillation flask and heated using a heating mantle, maintaining the temperature between 85 and 90 °C. This temperature range was strategically selected to recover light volatile compounds, particularly low molecular weight, high-value-added molecules, as described by Pinheiro Pires [84]. The crude pyroligneous liquid was subjected to fractional distillation in a borosilicate glass flask maintained at 85–90 °C. The process aimed to recover the light volatile compounds present in the mixture. The distillation was carried out under atmospheric pressure and continued until no further evaporation was visually observed. The distilled fraction was continuously collected via condensation and stored for subsequent analysis. At the end of the process, the residue remaining in the flask—rich in less volatile and tar-like components—was separated and also stored.

The yields of the distillate and the residue were calculated gravimetrically, based on the initial mass of the crude pyroligneous liquid and the final mass of each collected fraction. Both the crude and distilled fractions were later used in germination and plant growth assays, as well as for chemical characterization, including FTIR and GC–MS analyses.

### 3.7. Chemical Analysis of Liquid Fractions

The crude pyroligneous liquid, the distilled fraction, and the residual tar were analysed to identify the main components by gas chromatography–mass spectrometry (GC-MS), using a Shimadzu QP2010 PLUS system (Shimadzu Corporation, Tokyo, Japan). A 1 μL sample was injected with a split ratio of 50. The injector temperature was set to 220 °C, and helium was used as the carrier gas at a column flow of 0.99 mL/min. The detector temperature was set to 230 °C. The oven temperature program started at 60 °C (3 min hold), increased at 5 °C/min to 240 °C, and was held for 10 min. A Carbowax capillary column was used for compound separation. Compounds were identified using the NIST05 library (National Institute of Standards and Technology), with a similarity index above 80%.

### 3.8. Antioxidant Activity of Liquid Fractions

The antioxidant activity of the liquid fractions was assessed using three methods: DPPH (2,2-diphenyl-1-picrylhydrazyl), FRAP (Ferric Reducing Antioxidant Power), and total phenolic content determined by the Folin–Ciocalteu method. All analyses were performed in triplicate.

#### 3.8.1. Determination of Antioxidant Activity by the DPPH Method

The antioxidant capacity was evaluated according to the method proposed by Brand-Williams et al. [85], with adaptations by Sánchez-Moreno et al. [76], which measures the extract’s efficiency in scavenging the DPPH radical. Briefly, 0.1 mL of the sample was added to 3.9 mL of DPPH solution (60 μM in methanol) and kept in the dark for 45 min. Absorbance was measured at 515 nm using methanol as a blank.

The standard curve was constructed using DPPH solutions ranging from 10 to 60 μM. The EC_50_ value (μg/mL) was calculated as the concentration required to reduce 50% of the initial absorbance of the DPPH radical, according to Equation (1):

(1) $$\mathrm{EC}50=\frac{((\frac{\mathrm{ABS}}{2})-0.0116)}{0.0073}\;\times \;\frac{394.3}{1000}$$

where the values 0.0116 and 0.0073 correspond to the slope and intercept, respectively, of the linear regression equation obtained from the DPPH standard curve (10 to 60 µM). These parameters were used to convert absorbance values into µM of antioxidant equivalents, allowing the estimation of the EC_50_ values for each sample. The factor 394.3 corresponds to the molecular weight of DPPH, and the division by 1000 converts the units to µg/mL.

#### 3.8.2. Determination of Antioxidant Capacity by the FRAP Method

The antioxidant capacity using the FRAP method (Ferric Reducing Antioxidant Power) was determined according to the procedure described by Pulido et al. [86], evaluating the reduction of Fe^3+^ to Fe^2+^. The FRAP reagent was prepared by mixing TPTZ (10 mmol/L in 40 mmol/L HCl), FeCl_3_·6H_2_O (20 mmol/L), and acetate buffer (300 mmol/L, pH 3.6) in a 1:1:10 ratio.

The reaction was initiated by adding 3 mL of the FRAP reagent to 0.1 mL of the sample, followed by incubation for 5 min. Absorbance was measured at 593 nm using gallic acid (0.55 mM) as a standard and distilled water as a blank. Antioxidant activity was expressed as μM Fe^2+^/g of sample, calculated from the standard curve equation using ferrous sulphate.

#### 3.8.3. Quantification of Total Phenolic Compounds by the Folin–Ciocalteu Method

The total phenolic content was determined using the Folin–Ciocalteu method Singleton et al. [87]. For this, 250 μL of the methanol-extracted supernatant was mixed with 4 mL of distilled water and 250 μL of Folin–Ciocalteu reagent (1:1). After 5 min of reaction, 0.5 mL of sodium carbonate (Na_2_CO_3_, 7%) was added, and the samples were left to stand for 2 h in the dark. Absorbance was measured at 725 nm, and the phenolic concentration was determined using a gallic acid standard curve (10 to 100 μg/mL). Results were expressed as mg of gallic acid equivalents (GAE) per mL of extract.

### 3.9. Bioactivity Assay

The bioactivity tests were performed using both the crude and distilled pyroligneous liquid fractions at different concentrations (0.01% and 0.25%); higher concentrations were tested but showed an inhibitory effect on the seeds. The bio-stimulant potential of the pyroligneous liquid was evaluated using lettuce (*Lactuca sativa*) and arugula (*Eruca sativa*) seeds, which were placed in Petri dishes containing filter paper moistened with the test solutions. A volume of 4 mL of each prepared solution was added, and the dishes were incubated in a growth chamber (BOD) at 22 °C for 48 h, under controlled humidity and lighting conditions. Each treatment was carried out in triplicate, with a specific number of seeds per dish, defined according to the species used.

After 48 h of incubation, the seeds were assessed for germination index, root length, relative elongation and vigour index. The index germination (IG) was calculated to assess the combined effects of germination percentage and root elongation in relation to the control. The formula used was as follows:

$$\mathrm{IG}=\left(\frac{\mathrm{GT}}{\mathrm{GC}}\;\times \;\frac{\mathrm{LT}}{\mathrm{LC}}\right)\;\times \;100$$

where: GT and LT are the germination percentage and the average root length of the treatment, respectively, and GC and LC are the values from the control group. The control was standardized as 100%. GI values above 100% indicate stimulatory effects, while values below 100% indicate inhibitory responses.

In addition, the vigour index was determined using the following equation:

$$\mathrm{IV}\;\left(\%\right)=\frac{\left(\mathrm{TG}\left(\%\right)\;\times \;\mathrm{AR}\left(\%\right)\right)}{100}\;\times \;100$$

It was interpreted as follows: a vigour index (VI) above 100% indicated a bio-stimulant effect, between 80–100% indicated a neutral effect, and below 80% suggested potential inhibition.

All germination and growth experiments were conducted in triplicate, using three biological replicates per treatment. The results were analysed using one-way ANOVA followed by Tukey’s post hoc test, with a significance level set at *p* < 0.05. Statistical analyses were performed using OriginPro 2022.

## 4. Conclusions

Slow pyrolysis of rice husk proved to be a viable route for obtaining biochar and pyroligneous liquid with distinct properties. The distillation of the liquid fraction reduced its acidity and promoted the concentration of bioactive compounds, such as phenols and ketones, resulting in greater chemical stability and antioxidant activity.

The distilled fraction, applied at 0.1%, showed a significant bio-stimulant effect on the growth of lettuce and arugula seedlings, while higher concentrations were phytotoxic. The results indicate that pyroligneous liquid, especially in its distilled form, has potential as a natural agricultural input, provided it is applied at appropriate doses.

The combination of chemical composition, thermal behaviour, and bioactivity results confirms the potential of distilled pyroligneous liquid from rice husk as a selective and functional bioproduct with applications in sustainable agriculture and natural antioxidant systems.

## 5. Patents

The following patent resulted from the research presented in this manuscript:

Goularte, M.P.; Sousa, A.F.; Romano, L.R.W.; Pires, W.F.; Gatto, D.A. Sustainable Rooting and Fortifying Agent Based on Pyroligneous Liquid for Efficient Root Stimulation and Plant Strengthening. Brazilian Patent BR 10 2024 023791, filed on 14 November 2024, INPI—Brazilian National Institute of Industrial Property.

## Figures and Tables

**Table 1:** Compounds identified in the fractions of crude pyroligneous liquid (LB) and rice husk distillate (LD), determined by GC-MS. The data is expressed as a percentage of relative area (%). (–) indicates that the compound was not detected in the respective fraction.

Compound	Formula	Crude (%)	Distilled (%)
Pyridine	C_5_H_5_N	0.42	–
2-Methylpyridine	C_6_H_7_N	0.08	–
Formamide	CH_3_NO	0.06	–
1,6-Heptadien-4-ol	C7H_12_O	0.06	–
Butanoic acid, 2-propenyl ester	C_7_H_12_O_2_	0.11	0.06
2-Butanone, 3-hydroxy-	C_4_H_8_O_2_	0.46	0.75
2-Propanone, 1-hydroxy-	C_3_H_6_O_2_	11.69	14.5
Furan, 2-methyl-	C_5_H_6_O	1.09	2.09
2-Cyclopenten-1-one, 2-methyl-	C_6_H_8_O	0.17	–
1-Hydroxy-2-butanone	C_4_H_8_O_2_	1.33	51.53
Acetic acid	C_2_H_4_O_2_	52.84	–
Furfural	C_5_H_4_O_2_	1.37	2.92
2-Propanone, 1-(acetyloxy)-	C_5_H_8_O_3_	2.18	–
Formic acid	CH_2_O_2_	2.85	2.97
2,5-Hexanedione	C_6_H_10_O_2_	0.42	–
2-Cyclopenten-1-one, 3-methyl-	C_6_H_8_O	0.37	–
Propanoic acid	C_3_H_6_O_2_	2.49	3.96
Butyrolactone	C_4_H_6_O_2_	0.79	0.36
Butanoic acid	C_4_H_8_O_2_	0.69	–
3-Hexen-2-one, 3-methyl-	C_7_H_12_O	0.11	0.27
2-Furanmethanol	C_5_H_6_O_2_	0.31	–
2(5H)-Furanone, 3-methyl-	C_5_H_6_O_2_	0.13	–
2(5H)-Furanone	C_4_H_4_O_2_	0.34	–
Crotonic acid	C_4_H_6_O_2_	0.26	0.30
1,2-Cyclopentanedione, 3-methyl-	C_6_H_8_O_2_	1.5	0.55
Phenol, 2-methoxy-	C_7_H_8_O_2_	2.5	3.46
2-Cyclopenten-1-one, 3-ethyl-2-hydroxy-	C_7_H_10_O_2_	0.18	–
Phenol, 2-methoxy-4-methyl-	C_8_H_10_O_2_	1.4	1.4
Carbamic acid, phenyl ester	C_7_H_7_NO_2_	2.99	3.42
Phenol, 4-ethyl-2-methoxy-	C_9_H_12_O_2_	0.28	–
Cyclopropyl carbinol	C_4_H_8_O	2.57	–
Phenol, 2-methyl-	C_7_H_8_O	0.72	1.04
2-Hydroxy-gamma-butyrolactone	C_4_H_6_O_3_	0.34	–
1,6-Anhydro-beta-D-glucopyranose	C_6_H_10_O_5_	1.35	–
4-Pentenal	C_5_H_8_O	–	0.14
Butanal, 3-methyl-	C_5_H_10_O	–	0.14
1,4-Butanediol, 2,3-bis(methylene)-	C_6_H_10_O_2_	–	0.82
1,2-Epoxy-3-propyl acetate	C_5_H_8_O_3_	–	2.09
1-Octen-3-yl-acetate	C_10_H_18_O_2_	–	0.65
3-Pentanone, 2-methyl-	C_6_H_12_O	–	0.17
Hexanoic acid	C_6_H_12_O_2_	–	0.94
N-carbobenzyloxy-l-tyrosyl-l-valine	C_22_H_26_N_2_O_6_	–	0.44
Phenol, 3-ethyl-	C_8_H_10_O	0.53	0.76

**Table 2:** Comparison of antioxidant activity (DPPH and FRAP) and total phenolic content of crude pyroligneous liquid, tar, and distillate fractions.

Method	Crude	Tar	Distillate
**DPPH (EC_50_ µg/mL)**	7.881 ± (3.43) ^b^	216.78 ± (40.10) ^a^	10.98 ± (2.30) ^b^
**FRAP (µM Fe^2+^/g)**	7.62 ± (3.38) ^b^	6.08 ± (0.25) ^b^	23.40 ± (1.14) ^a^
**Total phenolics (mg/mL)**	713.72 ± (6.17) ^c^	729.25 ± (7.55) ^c^	708.38 ± (1.37) ^c^

Means followed by the same letter do not differ horizontally from each other according to Tukey’s test (*p* < 0.05).

## Data Availability

The data presented in this study are available on request from the corresponding author.

## Abbreviations

The following abbreviations are used in this manuscript:

DPPH	2,2-Diphenyl-1-picrylhydrazyl
EC50	Half Maximal Effective Concentration
EAG	Gallic Acid Equivalent
FRAP	Ferric Reducing Antioxidant Power
LD	Distilled Pyroligneous Liquid
LB	Crude Pyroligneous Liquid
TG	Thermogravimetric Analysis
Fe^2+^	Ferrous Ion
